# Association between 5-min Apgar score and attention deficit hyperactivity disorder: a Scotland-wide record linkage study of 758,423 school children

**DOI:** 10.1186/s12888-023-05217-6

**Published:** 2023-10-31

**Authors:** Jecintha J. Bala, Joel D. Bala, Jill P. Pell, Michael Fleming

**Affiliations:** 1 School of Health and Wellbeing, University of Glasgow, Glasgow, 90 Byres Road, G12 8TB UK; 2 Hospital At Home, NHS Central London Community HealthCare Trust, London, SW17 9SH UK

**Keywords:** Apgar score, Attention deficit disorder with hyperactivity, Cohort studies, Education, Medical record linkage

## Abstract

**Background:**

Attention-deficit hyperactivity disorder (ADHD) affects around 1 in 20 children and is associated with life-long sequelae. Previous studies of the association between Apgar score and ADHD have reported inconsistent findings.

**Methods:**

Record linkage of maternity, prescribing and school pupil census databases was used to conduct a population e-cohort study of singleton children born in Scotland and attending school in Scotland at any point between 2009 and 2013. Binary logistic regression analysis was used to investigate the association between 5-min Apgar score and treated ADHD adjusting for sociodemographic and maternity confounders.

**Results:**

Of the 758,423 children, 7,292 (0.96%) received ADHD medication. The results suggested a potential dose–response relationship between Apgar score and treated ADHD independent of confounders. Referent to an Apgar score of 10, risk of treated ADHD was higher for scores of 0–3 (adjusted OR 1.76, 95% CI 1.32–2.34), 4–6 (adjusted OR 1.50, 95% CI 1.21–1.86) and even 7–9 (adjusted OR 1.26, 95% CI 1.18–1.36) which are traditionally considered within the normal range.

**Conclusions:**

In addition to reinforcing the need to maximise Apgar score through good obstetric practice, the findings suggest that Apgar score may be useful in predicting future risk of ADHD and therefore facilitating early diagnosis and treatment.

## Introduction

Attention-deficit hyperactivity disorder (ADHD) is thought to affect around 5% of school-aged children globally [1], however estimates vary due to differences in the demographics of study populations and the diagnostic criteria applied [2, 3]. Worldwide, the prevalence of ADHD is between 2 and 7%, with a mean of 5% [4]. Based on administrative data, Holden estimated a childhood UK prevalence of 0.55% (0.93% in boys, and 0.15% in girls [5]. Additionally, a more recent retrospective cohort study in the UK indicated that the childhood prevalence of ADHD was 1.8% among boys and 0.4% among girls [6]. Due to increased knowledge, recognition, and diagnosis, the prevalence of ADHD amongst children in the UK has increased in recent years [5, 6].

The condition is generally characterised by consistent inattention, which may be accompanied with or without hyperactivity or impulsivity, but inattention is not present in a minority of cases [7]. Comorbidity is common and includes: dyslexia, low self-esteem, reading, writing and executive function challenges, mood disorders, learning disability, substance abuse, conduct and oppositional defiant disorders, Tourette syndrome, coordination difficulties, and autism spectrum disorder [8]. Symptoms persist into adulthood for 15–65% of affected children [9] and ADHD is associated with significant long-term social and economic consequences [7], including academic underachievement [10], challenges with careers and relationships [11], and criminality [12].

The Apgar score is routinely recorded on all newborn infants, 1- and 5-min following birth, irrespective of place of delivery. It was originally developed in 1952 to identify those in need of respiratory support [13]. Scores of 0–2 are allocated to each of five domains—colour, heart rate, reflexes, muscle tone, and respiration – and summated. The Apgar score is the sum of the sub-scores for each component, producing an overall score of 0–10. Overall scores in the range 7–10 are considered normal, 4–6 are considered low, and 0–3 very low [14]. Apgar scores taken at 1- minute frequently reflect acute perinatal events affecting oxygen supply, while 5-min Apgar scores measure the effectiveness of resuscitation. The Apgar score quantifies clinical manifestations of neonatal depression, such as cyanosis or pallor, bradycardia (reduced heart rate), impaired reflex response to stimulation, flaccidity of the muscles, and apnoea or gasping respirations [15]. In addition, it offers a reliable and practical way to report on the new-born baby’s condition immediately after birth and the baby's reaction to resuscitation, if necessary. Since its creation five decades ago, the Apgar score has become widely accepted as a measure of all new-born's viability and is widely utilized as an index of all infant's vitality shortly after birth [13].

Whilst not developed as a risk prediction tool, low Apgar score has nonetheless been shown to be associated with adverse neurological outcomes, including cerebral palsy (https://www.ncbi.nlm.nih.gov/books/NBK470569/#article-17763.s2). APGAR score itself is not mechanistically or causally associated with ADHD. Rather, it is a measure of the neonate’s overall status at that point in time and, therefore, a proxy measure of underlying biological mechanisms that may be causal of ADHD. For example, prenatal and intrapartum hypoxia are known to impact both APGAR score [16] and neurodevelopmental outcomes such as cerebral palsy, autism, and cognitive dysfunction [17]. However, the Apgar score in isolation cannot confirm hypoxia or its consequences [18]. Further, evidence of anomalies in neuroimaging [19] and low umbilical arterial blood gas results could be indicative of ADHD [20]. Furthermore, it has been suggested that a low Apgar score may result in neonatal hypoxia [21], which may have an impact on the dopamine-related neurodevelopmental pathways that relate to ADHD. Dopamine, a crucial neurotransmitter involved in memory, emotion, attention, behaviour, cognition, learning, sleep, and arousal, is low in those with ADHD [22]. Dopamine is produced by the basal ganglia of which perinatal anoxia has been demonstrated in animal experiments to alter mesocortical dopamine function and elevate hyperactivity [23]. Also, the dopaminergic pathways, which are thought to be crucial in the pathophysiology of ADHD, depend on the basal ganglia, which are highly susceptible to hypoxia [24]. Although the Apgar score can indicate infant hypoxia, it cannot prove it [18] since other factors, such as prematurity, low birthweight, maternal illnesses, and toxins, can also affect it [25, 26]. Therefore, a number of pathways may be involved in the association between low Apgar scores and ADHD.

Previous studies examining the relationship between 5-min Apgar score and ADHD have been heterogeneous in their methods and have produced conflicting results. Some cohort studies have reported a dose–response relationship between 1-min [27] and 5-min [28] Apgar scores and ADHD in childhood, with ADHD risk being higher even for Apgar scores of 7–8 compared to 9–10. One population cohort study reported a dose–response relationship with increasing risk of ADHD in adulthood associated with Apgar scores 0–3 and 4–6 when compared to scores of 7–9 [29]. Other studies have reported mixed or negative results. In a nested case–control study, 1-min Apgar scores less than 7 were associated with higher odds of ADHD compared with scores of 7–10 [30]. However, there was no association with 5-min Apgar score. Similarly, a population-based study, conducted in Western Australia, found no association between low 5-min Apgar score and ADHD [31]. There is some evidence that Apgar score may also be associated with ADHD severity. In a cohort of children with ADHD, symptom severity, based on the externalising scale of the Child Behaviour Checklist and the Diagnostic and Statistical Manual of Mental Disorders (DSM-IV) hyperactivity symptoms count, was higher among those who had a 1-min Apgar score of 1–6 than those with Apgar scores of 7–10 [32].

Conflicting results from previous studies exist due to inconsistencies around adjustment for confounders and categorisation of Apgar score. Many previous studies have not adjusted for important factors such as maternal smoking status, sex, ethnicity, gestational age at delivery, birth-weight centile, mode of delivery and socioeconomic deprivation [27, 30] whilst others have used small sample sizes [30, 32, 33]. In this large-scale, retrospective population cohort study, we investigate the association between 5-min Apgar score and treated ADHD in childhood using linked health and education data on all children attending school in Scotland between 2009 and 2013. The study overcomes previous methodological limitations by utilising granular data covering the whole of Scotland and adjusting for a range of sociodemographic and obstetric confounders.

## Methods

### Data sources

Three Scotland-wide databases were linked at the individual level. The Scottish Exchange of Educational Data (ScotXed) provided data from the School Pupil Census which is conducted annually at the beginning of the academic year (September) and covers all local authority run primary, secondary, and special schools in Scotland. Public Health Scotland (PHS) provided data from the Prescribing Information System (PIS) and Scottish Morbidity Record 02 (SMR02). The PIS database covers all medications prescribed and dispensed in the community in Scotland, by community pharmacies or primary healthcare providers. The SMR02 maternity record collects data on pregnant women receiving inpatient or day case care and includes information at delivery pertaining to mother and child.

### Definitions and inclusion criteria

**Fig. 1:**
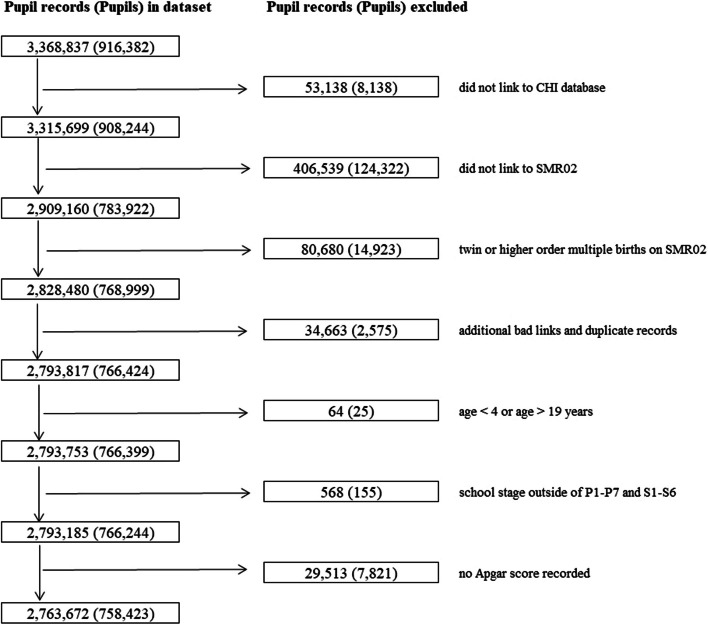
Flow diagram illustrating the number of pupils and pupil records included and excluded from the cohort at each stage of data cleaning

Our cohort comprised all children born from singleton pregnancies in Scotland and attending a local authority run primary, secondary, or special school at some point between 2009 and 2013. The cohort included children born in years 1991 through to 2009. Children could attend school in multiple years across the study period and could enter or leave the cohort at any point during this time. The number of observations per child ranged from a minimum of one observation (if they left school in 2009 or started school in 2013) to five observations (if they attended school across all years). The study was limited to children born from singleton pregnancies since it was impossible to be certain that the correct child had been linked in the case of multiple births of same-sex offspring. Children with no recorded Apgar score and children recorded as attending school at < 4 years of age or > 19 years of age were excluded. The number of pupils and pupil records omitted at each stage of data cleaning is summarised in Fig. 1.

Consistent with our previous work using the same linked cohort [10, 34, 35], treated ADHD was defined as being present in a given academic year if there had been at least one dispensed prescription during that academic year of a medication authorised only for this condition: methylphenidate, dexamphetamine, atomoxetine, or lisdexamphetamine. 5-min Apgar score was obtained from the SMR02 record and categorised as 0–3, 4–6 and 7–9, with 10 as the referent category. Maternal age at delivery, parity, maternal smoking during pregnancy, gestational age at delivery, mode of delivery, sex- and gestation-specific birthweight centiles and postcode of residence at the time of delivery were all obtained from the SMR02 record. History of smoking at any point during the current pregnancy is ascertained by midwives and recorded in the SMR02 record. Mothers are recorded as having smoked during pregnancy even if they stopped before confirmation of pregnancy but after their last menstrual cycle. Postcode of residence was used to derive the Scottish Index of Multiple Deprivation 2012 (SIMD) (http://www.gov.scot/Topics/Statistics/SIMD/BackgroundMethodology) from aggregated Census data (for median populations of 769 residents) on 38 markers across seven domains: income, occupation, health, accommodation, geographic access, offences, education, and abilities and competence. Pupil’s age, sex, and ethnicity were obtained from the School Pupil Census.

### Statistical analyses

The characteristics of children were summarised by Apgar score category using frequency and percentage and compared using χ^2^tests for trend and association and Spearman rank correlation. Binary logistic regression analysis was used to investigate the association between Apgar score and ADHD, using an Apgar score of 10 as the referent category. Because children had repeated outcome observations obtained across multiple census years, generalized estimating equations (GEE) were used to analyse the association between Apgar scores and ADHD within this longitudinal cohort. Using GEE models accounted for relationships between repeated observations relating to the same pupil throughout different census years [36]. Diverse correlation structures were compared using the user-written quasi-likelihood under the independence model criterion (QIC) statistic [37]. The most suitable correlation structure had the lowest trace QIC. For these data, an independent correlation structure was the most appropriate. The association between categorised Apgar score and ADHD was investigated using GEE analyses with an independent correlation structure, a binomial distribution, and a logit link function. Confidence intervals were produced using cluster-robust standard errors to account for correlations in the data.

The model was run univariately, followed by sequential multivariate models adjusting firstly for sociodemographic factors (age, sex, deprivation, and ethnicity) then also for obstetric factors (sex-gestation-specific birthweight centile, parity, mode of delivery, maternal age, gestational age, and maternal smoking status during pregnancy) and year of birth. The latter variable was to account for changes in Apgar score and changes in treatment of ADHD over time. Statistical analyses were undertaken using Stata MP, version 14.1 (StataCorp).

## Results

Of the 758,423 eligible children with valid data on Apgar score, 7,292 (0.96%) received treatment for ADHD in the follow up period which ranged from 1 year through to 18 years (median follow up 9 years). Overall, 3,709 (0.49%) children had Apgar scores of 0–3, 7,302 (0.96%) had scores of 4–6, 625,299 (82.45%) had scores of 7–9, and 122,113 (16.10%) scored 10. Children with lower Apgar scores were more likely to be treated for ADHD. Children with the lowest Apgar scores (0–3) were more likely to be male, deprived, of white ethnicity, have a mother aged 25–29 years who smoked and was multiparous, be born preterm, have a lower birthweight centile, and be born via emergency Caesarean section (Table 1). The variables with the greatest amount of missing data were smoking during pregnancy (9.42%), and pupil ethnicity (1.79%). However, missing values for those variables, as well as missing data for mode of delivery (0.02%), were analysed as “unknown” categories and included in all analyses to minimise loss of records. The remaining variables were ordinal categories therefore including missing data as unknown categories did not make sense. However, these variables had lower levels of missing values: parity (0.57%), maternal deprivation quintile (0.14%), birthweight centile (0.09%), gestational age (0.05%) and maternal age (< 0.01%). Therefore, we did not deem multiple imputation to be necessary and instead used complete case analyses. There were no missing data for pupil sex or pupil age. All the children had valid ADHD prescribing, because those who had not linked to healthcare data were omitted at the data cleaning stage (Fig. 1).

**Table 1:** Characteristics of schoolchildren by Apgar score

	Overall		Apgar 0–3		Apgar 4–6		Apgar 7–9		Apgar 10		*P*-value
	*N* = 758,423		*N* = 3,709		*N* = 7,302		*N* = 625,299		*N* = 122,113		
	n	%	n	%	n	%	n	%	n	%	
Treated for ADHD											
yes	7,292	0.96	64	1.73	110	1.51	6,196	0.99	922	0.76	< 0.001 ^1^
no	751,131	99.04	3,645	98.27	7,192	98.49	619,103	99.01	121,191	99.24	
Sex											
male	386,152	50.92	1,895	51.09	4,101	56.16	319,544	51.1	60,612	49.64	< 0.001 ^1^
female	372,271	49.08	1,814	48.91	3,201	43.84	305,755	48.9	61,501	50.36	
Parity											
0	342,225	45.35	1,598	43.18	3,995	55.02	282,070	45.35	54,562	44.83	< 0.001 ^3^
1	261,573	34.67	1,218	32.91	2,005	27.61	215,989	34.73	42,361	34.8	
> 1	150,867	20	885	23.91	1,261	17.37	123,924	19.92	24,797	20.37	
missing	3,867		8		41		3,316		393		
Gestation (weeks)											
< 27	598	0.08	73	1.97	105	1.44	393	0.06	27	0.02	0.017 ^3^
27–40	560,029	73.84	2,950	79.54	5,328	72.97	460,147	73.59	91,604	75.02	
> 40	197,796	26.08	686	18.5	1,869	25.6	164,759	26.35	30,482	24.96	
Sex- gestation-specific birthweight centile											
1–3	31,105	4.11	240	6.54	524	7.2	25,714	4.12	4,627	3.79	< 0.001 ^3^
4–10	67,859	8.96	388	10.57	818	11.24	55,893	8.95	10,760	8.82	
11–20	90,431	11.94	488	13.3	932	12.81	74,660	11.96	14,351	11.76	
21–80	445,729	58.84	2,021	55.07	3,900	53.59	367,326	58.82	72,482	59.4	
81–90	64,680	8.54	270	7.36	542	7.45	53,508	8.57	10,360	8.49	
91–97	40,803	5.39	155	4.22	357	4.91	33,590	5.38	6,701	5.49	
98–100	16,870	2.23	108	2.94	205	2.82	13,815	2.21	2,742	2.25	
missing	946		39		24		793		90		
Mode of delivery											
cephalic vaginal	510,766	67.36	2,276	61.36	3,842	52.62	425,279	68.01	79,369	70	< 0.001 ^2^
assisted vaginal	91,189	12.03	301	8.12	866	11.86	76,118	12.17	13,904	11.39	
breech vaginal	2,172	0.29	77	2.08	131	1.79	1,784	0.29	180	0.15	
elective Caesarean section	57,787	7.62	191	5.15	252	3.45	45,504	7.28	11,840	9.7	
emergency Caesarean section	96,351	12.71	853	23	2,211	30.28	76,484	12.23	16,803	13.76	
missing	156		11		0		130		17		
Maternal smoking											
No	488,991	72.37	2,111	67.32	4,523	69.81	405,171	72.62	77,186	71.42	< 0.001 ^1^
Yes	186,646	27.63	1,025	32.68	1,956	30.19	152,780	27.38	30,885	28.58	
missing	82,786		573		823		67,348		14,042		
Pupil ethnicity											
white	718,084	96.24	3,525	95.04	6,950	95.18	593,811	94.96	113,798	93.19	< 0.001 ^2^
Asian	17,566	2.35	77	2.08	139	1.9	12,930	2.07	4,420	3.62	
black	1,893	0.25	12	0.32	18	0.25	1400	0.22	463	0.38	
mixed	6,616	0.89	35	0.94	63	0.86	5,538	0.89	980	0.8	
other	1,962	0.26	4	0.11	16	0.22	1,653	0.26	289	0.24	
missing	12,302		56	1.51	116	1.59	9,967	1.59	2,163	1.77	
Maternal age (years)											
< = 24	207,511	27.36	1,025	27.64	2,188	29.96	170,574	27.28	33,724	27.62	< 0.001 ^3^
25–29	222,491	29.34	1,133	30.55	2,170	29.72	185,764	29.71	33,424	27.37	
30–34	214,856	28.33	1,007	27.15	1,869	25.6	177,171	28.33	34,809	28.51	
> = 35	113,553	14.97	544	14.67	1,075	14.72	91,778	14.68	20,156	16.51	
missing	12		0		0		12		0		
Deprivation quintile											
1 (most deprived)	203,814	26.94	1,047	28.36	2,122	29.14	162,494	26.05	38,151	31.3	< 0.001 ^3^
2	160,657	21.23	821	22.24	1,594	21.89	131,098	21.02	27,144	22.27	
3	139,443	18.43	671	18.17	1,342	18.43	116,539	18.68	20,891	17.14	
4	130,100	17.19	601	16.28	1,195	16.41	109,180	17.5	19,124	15.69	
5 (least deprived)	122,630	16.21	552	14.95	1,030	14.14	104,485	14.14	16,563	13.59	
missing	1,779		17		19		1,503		240		

*N* Number of pupils

^1^*P*-values to compare Apgar score against ADHD, sex and maternal smoking produced using Chi square test for trend

^2^*P*-values to compare Apgar score against mode of delivery and pupil ethnicity produced using Chi square test for association

^3^*P*-values to compare Apgar score against parity, gestation, birthweight centile, maternal age, and deprivation quintile produced using Spearman rank correlation

After fully adjusting for sociodemographic and maternity confounders, lower Apgar score was associated with higher risk of receiving ADHD medication and, whilst the confidence intervals overlapped, there was evidence of a potential dose–response relationship. Compared to those with an Apgar score of 10, the increase in risk was highest among children with an Apgar score of 0–3 (odds ratio (OR) 1.76, 95% CI 1.32–2.34), and was also significantly higher among children with an Apgar score of 4–6 (OR 1.50, 95% CI 1.21–1.86) and among children with an Apgar score of 7–9 (OR 1.26, 95% CI 1.18–1.36) (Table 2).

**Table 2:** Binary logistic regression analysis of the associations between Apgar score and treated ADHD

		Univariate (*N* =2,763,672[758,423])			Multivariate 1^a^ (*N* = 2,757,679[756,644])			Multivariate 2^b^ (*N* = 2,739,396[751,946])		
Variables	Categories	Odds ratio	95% CI	*P* value	Odds Ratio	95% CI	*P* value	Odds Ratio	95% CI	*P* value
Apgar score	0–3	2.24	1.71–2.94	< 0.001	2.33	1.77–3.06	< 0.001	1.76	1.32–2.34	< 0.001
	4–6	1.92	1.55–2.37	< 0.001	1.79	1.45–2.21	< 0.001	1.50	1.21–1.86	< 0.001
	7–9	1.30	1.21–1.39	< 0.001	1.34	1.25–1.44	< 0.001	1.26	1.18–1.36	< 0.001
	10 (ref)	-	-	-	-	-	-	-	-	-
Sex	male (ref)				-	-	-	-	-	-
	female				0.18	0.17–0.20	< 0.001	0.18	0.17–0.19	< 0.001
Deprivation	1 = most deprived				2.98	2.72–3.27	< 0.001	1.70	1.54–1.87	< 0.001
	2				2.31	2.09–2.54	< 0.001	1.55	1.40–1.71	< 0.001
	3				1.70	1.53–1.88	< 0.001	1.32	1.19–1.46	< 0.001
	4				1.30	1.16–1.45	< 0.001	1.15	1.03–1.28	0.016
	5 = least deprived (ref)				-	-	-	-	-	-
Pupil ethnicity	white (ref)				-	-	-	-	-	-
	Asian				0.14	0.09–0.22	< 0.001	0.15	0.10–0.23	< 0.001
	black				0.22	0.09–0.56	0.001	0.30	0.12–0.74	0.009
	mixed				0.61	0.44–0.85	0.004	0.64	0.46–0.89	0.008
	other				1.00	0.67–1.50	0.984	1.01	0.67–1.51	0.977
	unknown				1.33	1.17–1.51	< 0.001	1.31	1.15–1.49	< 0.001
Pupil age	< 11 (ref)				-	-	-	-	-	-
	11–14				1.37	1.32–1.43	< 0.001	1.08	1.03–1.13	0.001
	> 14				1.00	0.95–1.05	0.951	0.71	0.66–0.76	< 0.001
Maternal age	less than 25							1.69	1.58–1.80	< 0.001
	25–29 (ref)							-	-	-
	30–34							0.77	0.71–0.83	< 0.001
	35 and over							0.69	0.62–0.76	< 0.001
Estimated gestation								0.94	0.93–0.95	< 0.001
Birthweight centile	1–3							1.43	1.28–1.59	< 0.001
	4–10							1.24	1.14–1.35	< 0.001
	11–20							1.14	1.06–1.23	0.001
	21–80 (ref)							-	-	-
	81–90							0.91	0.82–1.00	0.042
	91–97							0.95	0.85–1.07	0.395
	98–100							0.97	0.82–1.15	0.716
Smoked during pregnancy	No (ref)							-	-	-
	Yes							1.65	1.56–1.75	< 0.001
	Unknown							1.09	1.00–1.19	0.047
Parity	none (ref)							-	-	-
	one child							1.09	1.03–1.16	0.004
	two or more children							1.52	1.42–1.63	< 0.001
Mode of delivery	spontaneous vaginal delivery (ref)							-	-	-
	cephalic							1.79	1.59–2.03	< 0.001
	assisted vaginal delivery							1.06	0.98–1.16	0.155
	breech delivery							0.76	0.49–1.20	0.247
	elective caesarean section							1.13	1.02–1.25	0.017
	emergency caesarean section							1.13	1.04–1.22	0.002
	other delivery							0.92	0.12–6.88	0.933
Year of Birth								0.95	0.95–0.96	< 0.001

*CI* Confidence interval, *N* Number of records [Number of pupils] included in analysis

^a^adjusted for sex, ethnicity, age, and deprivation quintile

^b^also adjusted for maternal age, gestation, birthweight centile, maternal smoking, parity, mode of delivery and year of birth

## Discussion

The study findings demonstrated a significant association between 5-min Apgar score and treated ADHD that was independent of sociodemographic and maternity confounders, with evidence of a potential dose–response relationship. Even children with Apgar scores of 7–9, which are generally interpreted as being within the normal range, had a significantly higher risk of ADHD than children who scored 10.

Previous studies have used varying approaches to categorising Apgar score. We used the categories recommended in the Neonatal Encephalopathy and Neurologic Outcome report, which defines an Apgar score of 0–3 as low and 4–6 as moderately abnormal. Comparison of Apgar scores of 7–9 with 10 enabled us to demonstrate that the potential dose–response relationship extended across the whole spectrum of scores and that even slightly reduced scores carried increased risk. This finding is consistent with previous studies showing that, compared to an Apgar score of 10, Apgar scores of 7–9 are associated with higher risk of neonatal morbidity and mortality, neurodevelopmental sequelae [38], autistic spectrum disorder [39], and problems with emotional and physical health [40, 41].

Previous studies have produced conflicting results as to whether 5-min Apgar score is associated with ADHD [28, 29] or is not [27, 31]. Many previous studies have treated Apgar score as a binary variable or only investigated low Apgar score however a few previous studies have examined whether there is evidence of a dose–response relationship [28, 42] across the spectrum and, consistent with our findings, demonstrated that Apgar scores below 10 but within the ‘normal’ range are nonetheless associated with increased risk of ADHD. Whilst they found a significant relationship, the effect sizes they obtained were inconsistent across studies. These differences can be partly explained by the method of ADHD ascertainment. To ascertain ADHD, Sucksdorff et al. [43] used a discharge register to identify cases. Whilst it was validated, there is still a possibility of misclassification bias and incomplete or incorrect information. It is equally possible that the authors missed some cases not included in the register. Schwenke et al. [30] used questionnaires which can often be affected by high rates of non-responders. Information was obtained from a caregiver or the parent with can also result in recall bias. Li et al. [28] and Halmoy et al. [29] utilised multiple data sources, including discharge registers, medical product statistics, information from clinicians, and informants' ascertainment of cases, potentially reducing information bias. However, like most secondary data, there is still potential for misclassification bias.

Choice of confounding variables, and methods used to adjust for them, varied across previous studies. Sucksdorff et al. [43] used logistic regression analysis to adjust for various confounders, including gestational age, weight for gestational age, maternal age and maternal socioeconomic status. They also adjusted for the mother's smoking status, alcohol intake, substance abuse during pregnancy, and history of maternal psychiatric diagnoses. Adjustments for maternal mental history and substance misuse were based on specialised public health care diagnoses. As a result, the authors could not adjust for ailments or personality traits treated in primary healthcare or private hospitals. This limitation is typical of observational studies where residual confounders are often present. While Sucksdorff et al. [43] adjusted for a wide range of confounders, Chandola et al. [27] only adjusted for sex in their study since most social data were not recorded in the Cardiff births register (CBR). However, the large control group enabled them to detect small but clinically significant differences between groups. Unlike Sucksdorff et al., [43] Chandola et al. [27] were limited by the amount of information in the register and resultant inability to adjust for a wide range of confounders.

Halmoy et al. [29] undertook their study adjusting for sex, mother’s age, maternal and paternal academic attainment, and parity. However, there were missing data on additional confounders, such as the history of psychiatry disorders, parents’ smoking habits, and the presence of other comorbid conditions. The confounders adjusted for were not as wide-reaching as in Surskdorff’s work [43]. Gustaffson et al., [44] like Halmoy et al., [29] used multivariable analyses to adjust for confounders, namely, year of birth, maternal age, maternal smoking, birthweight centile (birthweight for gestational age) and parity. However, the authors still lacked information on socioeconomic status, and like Halmoy et al., [29] did not have data on family history of mental disorders and abuse.

Mikkelsen et al. [16] and Li et al. [28] also used regression models to adjust for confounders. Li et al. [28] adjusted for sex, parity, gestational age, and low birth weight, family history of mental illness, maternal socioeconomic status, and smoking status during pregnancy using Cox regression. Mikkelsen et al. [16] used logistic regression, while Grizenko et al. [32] utilised a step-wise linear regression and adjusted for the same variables as Li et al. [28] except for gestational age and household history of mental ailments. Hanc et al. [33] in a case control study, matched the boys' age, socioeconomic factors, and parents' academic attainment.

The present study has several methodological strengths. The study sample of 758,423 children, was 25 times larger than any previous study addressing the same research question. We were able to adjust for a wide range of potential sociodemographic and maternal confounders including some not included in previous analyses: ethnicity, socioeconomic deprivation, child’s age, and mode of delivery. Inclusion was non-selective and country-wide, reducing the risk of bias and improving the generalisability of the results. Secondary analysis of routine databases obviated the possibility of reporting or recall bias. The grouping of Apgar scores in this study aligns with the recommendation by the Neonatal Encephalopathy and Neurologic Outcome report [19], projecting a more standardised and uniform way of categorising Apgar scores. Sub-categorising Apgar scores of 7–10 into 7–9 and 10, enabled us to examine whether it is safe to assume that any score below 10 is without risk.

As with all observational studies, causality cannot be automatically inferred from association.

However, our finding of a potential dose–response relationship is an important one. As with any observational research, there is potential for residual confounding. Specifically, we did not have access to data on family history of mental health disorders, relevant paternal factors like age, paternal history of ADHD, maternal substance abuse, and intra-uterine exposure to teratogens, and maternal history of ADHD. The annual School Pupil Census does not cover private schools, but these account for fewer than 5% of Scottish schoolchildren. With routine data, controlling completeness and accuracy is more difficult, however the databases used are all subject to regular quality control audits. Probabilistic matching was used to link education and health records however this method has been validated as 99% accurate for singletons [45]. The childhood prevalence of ADHD is around 5% in Scotland [46]. However, only 1% of children were receiving ADHD medication. The difference is likely to reflect our ascertainment method based on prescribed medication. Non-pharmacological treatment consisting primarily of parenting interventions that focuses on behavioural management is generally recommended and treatment is only reserved for those who meet certain criteria and are considered to have severe ADHD. Therefore, our ascertainment method has the limitation of potential misclassification bias whereby children with undiagnosed or milder untreated ADHD would not be ascertained and would be classed as condition free, whilst children classed as having ADHD would likely comprise those with more severe forms of the condition. This may have underestimated the strength of the relationship between Apgar score and ADHD. Unfortunately, we could not determine age of ADHD onset from our data as our observation period only started from 2009 onwards. Therefore, prescriptions for ADHD (our method of ascertainment) administered before this time were not available. Our study enabled us to ascertain children who 1. Were already on treatment for ADHD at the start of the study period (2009) or 2. Went on start treated during the study period (2009–2013). Age at commencement of treatment was only available for those in category 2 and age at formal diagnosis was not available for either.

## Conclusions

There was a significant, and potential dose–response, relationship between Apgar score and treated ADHD. In addition to reinforcing the need to maximise Apgar score through good obstetric practice, these findings suggest that Apgar score could be indicative of future risk of ADHD, therefore facilitating earlier diagnosis and treatment.

## Data Availability

The datasets generated and analysed during the study are not publicly available. All health data are owned by Public Health Scotland (https://www.publichealthscotland.scot), and all education data are owned by the Scottish Government (www.2.gov.scot/Topics/Statistics/ScotXed). Under the terms of our data access agreements with them we are not permitted to pass the data onto third parties. Interested researchers may apply at these sites for data access to health and education data by emailing phs.edris@phs.scot and ASU_schools_Data_Access@gov.scot respectively. The authors applied for permission to access, link, and analyse these data and undertook mandatory training in data protection, IT security and information governance. The study was approved by the National Health Service (NHS) Public Benefit and Privacy Panel and covered by a data processing agreement between Glasgow University and Public Health Scotland and a data sharing agreement between Glasgow University and ScotXed. The electronic Data Research and Innovation Service (eDRIS) within Public Health Scotland helped the authors obtain approvals, linked the data, and uploaded the final datasets into a secure analytical platform within the National Safe Haven for the researchers to analyse. The researchers did not receive any special privileges or access to the third-party data.
